# Spring resting egg production of the calanoid copepod, *Eurytemora affinis*, in a freshet-dominated estuary

**DOI:** 10.1093/plankt/fbae039

**Published:** 2024-07-20

**Authors:** Joanne Breckenridge, Evgeny Pakhomov

**Affiliations:** Department of Earth, Ocean and Atmospheric Sciences, University of British Columbia, Vancouver, BC V6T 1Z4, Canada; Department of Earth, Ocean and Atmospheric Sciences, University of British Columbia, Vancouver, BC V6T 1Z4, Canada; Institute of Oceans and Fisheries, University of British Columbia, Vancouver, BC V6T 1Z4, Canada

**Keywords:** dormancy, resting eggs, estuarine retention, Eurytemora affinis, Fraser River Estuary

## Abstract

Seasonal peaks in river discharge, such as snowmelt-dominated freshets, are predictable events that can have a large effect on flushing rates and salinity in estuaries. Resting eggs, which many coastal and estuarine copepods produce for overwintering or aestivation, could also serve to bridge predictable peaks in river discharge. We assessed the timing of resting egg production of the egg-carrying estuarine copepod, *Eurytemora affinis* (Poppe), in relation to river discharge in the Fraser River Estuary, Canada. Approximately 30 field-collected females were individually incubated on 12 occasions over the period February 2015–May 2016. *Eurytemora affinis* abundance and population structure were investigated from vertical net tow samples collected twice monthly to monthly. Resting eggs occurred primarily in May 2015 and May 2016 (6.5 and 9.2 eggs day^−1^, respectively), a month prior to peak flows, and the proportion of offspring that were resting eggs increased with river discharge. *Eurytemora affinis* reached a minimum abundance in July 2015, when the population was dominated by adults (86%). Resting egg production in *E. affinis* is typically considered an overwintering mechanism but we suggest that the ultimate driver of resting egg production in this population is avoidance of flushing and/or low salinities.

## INTRODUCTION

Estuarine endemic copepods face the persistent risk of advection from their required habitat (Ketchum, 1954). These taxa typically cannot survive fully marine waters making the consequences of advection from their estuary dire. These taxa live in an environment where net flow is out to sea, and yet, as planktons, they are unable to swim against a current. Nonetheless, estuarine endemic copepod species often reach high densities in this physically and chemically dynamic environment (Haertel and Osterberg, 1967; Heinle, 1972; Escaravage and Soetaert, 1995; Peitsch *et al.,* 2000; Bollens *et al.,* 2011), exploiting abundant food resources and, in turn, being preyed upon by other invertebrates and planktivorous fish (Winkler *et al.,* 2003; Strydom *et al.,* 2014).

The influence of advection risk in the evolution of estuarine endemic copepod species is evidenced in the suite of traits they possess that increase the likelihood of retention. Most pointed are the many examples of these copepod species controlling their vertical position in the water column to avoid being flushed from the estuary, including engaging in active vertical migration with the tides (Hough and Naylor, 1991; Kimmerer *et al.,* 1998; Ueda *et al.,* 2010; Chew *et al.,* 2015), aggregation near bottom during floods (Ueda *et al.,* 2004; Shang *et al.,* 2008) and having an epibenthic distribution (Sibert, 1981). Other traits that are common in estuarine endemic copepods that would promote retention include a short generation time (Miller, 1983; Mauchline, 1998) and possibly egg-carrying (Bayly, 1964; Breckenridge *et al.,* 2015). Some authors (Uye, 1980; Runge and Simard, 1990; Newton and Mitchell, 1999) have suggested that benthic resting eggs could help estuarine-endemic copepod populations avoid flushing. This idea is very intuitive. Dormancy is the common evolutionary response to a variable environment for short-lived organisms (Caceres, 1997) and dormant eggs in the sediment would allow pelagic species to bridge periods where losses due to flushing were greater than increases from reproduction. The production of benthic resting eggs appears common in estuarine copepods (Marcus and Boero, 1998; Holm *et al.,* 2018) and egg banks have been found in multiple estuaries (Marcus, 1991; Marcus *et al.,* 1994; Belmonte *et al.,* 1995; Newton and Mitchell, 1999; Katajisto, 2006; Glippa *et al.,* 2011; Berasategui *et al.,* 2013). The stage is well set; however, we were unable to find any evidence in the literature in support of this hypothesis. When reported for estuaries, the production of resting eggs is instead most often considered a mechanism to avoid periods outside an organism’s thermal tolerance (Holm *et al.,* 2018).

Knowledge of the timing of resting egg production may provide some clue as to its adaptive significance. When dormancy occurs in avoidance of a predictable event, an optimal timing for the switch to resting egg production would be favored over the continuous production of resting eggs (Hairston and Munns, 1984; Hairston and Bohonak, 1998); however, estuarine studies that investigate the timing of resting egg production under natural conditions are uncommon (Sullivan and McManus, 1986; Chen and Marcus, 1997; Castro-Longoria and Williams, 1999; Berasategui *et al.,* 2016). Seasonal peaks in river discharge, such as those that occur in snowmelt-dominated systems, are predictable events that can have a large effect on flushing rates in estuaries. In the Hopkins River Estuary, Australia, river discharge peaks late winter-early spring and the salt wedge is flushed from the estuary for a period of 1–3 months (Newton and Mitchell, 1999). Eggs from several abundant estuarine-endemic copepod species were hatched from estuarine sediments and the authors posited that these eggs likely served a role in retaining these populations (Newton and Mitchell, 1999). Indeed, benthic resting stages could be expected to be of greatest benefit to populations in estuaries that experience periods of high flushing. In common with the Hopkins River Estuary, the Fraser River Estuary (FRE), Canada, experiences extended periods where salt wedge incursion is greatly decreased due to high river flow. The FRE is subject to scouring freshets that occur late spring-early summer with snowmelt. During this period the salt wedge incursion does not extend far beyond the tidal flats at the mouth of the estuary and all salt is removed from the system on the greater ebb (Kostaschuk and Atwood, 1990; Jay *et al.,* 2007). Low zooplankton densities and the restriction of estuarine species to backwaters suggest that zooplankton production in the larger estuary is limited by low water residence time (Breckenridge *et al.,* 2020). That the FRE supports populations of estuarine endemic copepods, despite the freshwater conditions that prevail in the late spring and early summer (Breckenridge et al.2020) suggests the availability of some refuge which allows for the retention of these populations. To our knowledge, production of resting eggs prior to a period of high river discharge and low water residence time has never been reported in the literature.

This study assesses the timing of resting egg production of the calanoid copepod *E. affinis* (Poppe) in relation to peak river discharge in the FRE and is the first focused investigation of *E. affinis* life history in this estuary. *Eurytemora affinis* is an ecologically important species complex that occurs in estuaries throughout the Northern Hemisphere (Simenstad *et al.,* 1990; Lee, 2000; Winkler *et al.,* 2003; Dauvin and Desroy, 2005) and often reaches very high densities (Heinle and Flemer, 1975; Devreker *et al.,* 2010). Resting egg production appears to be common in *E. affinis* (Johnson, 1980; Ban, 1992; Madhupratap *et al.,* 1996; Marcus, 1996; Glippa *et al.,* 2011), though not necessarily ubiquitous (Choi and Kimmerer, 2009), and is believed to occur in autumn as a mechanism to avoid winter conditions (Glippa *et al.,* 2013; Holm *et al.,* 2018). *Eurytemora affinis* are egg-carrying species and subitaneous eggs are held until hatch (Mauchline, 1998; Glippa *et al.,* 2011). In the FRE, *E. affinis* abundance is at a minimum during the early summer, coincident with peak Fraser River discharge, and peaks in late summer and early autumn as discharge decreases (Breckenridge *et al.,* 2020). This annual pattern of abundance is in contrast to other estuarine systems where this species is at minimum abundance in the winter and peaks in the spring (Haertel and Osterberg, 1967; Heinle and Flemer, 1975; Sautour and Castel, 1995; Peitsch *et al.,* 2000; Kimmel and Roman, 2004; Lawrence *et al.,* 2004; Devreker *et al.,* 2010). Modeling of *E. affinis* population dynamics in the FRE suggested that the hatching of resting eggs was necessary to explain high observed post-freshet abundances (Breckenridge, 2022). We hypothesized that *E. affinis* in the FRE produce resting eggs in the spring to avoid advection from the estuary. The aims of this paper are to (1) provide estimates of egg production and release by *E. affinis* in the FRE, and (2) to interpret variation in egg release with respect to underlying patterns of abundance, population structure and environmental conditions.

## METHOD

### Study site

**Fig. 1:**
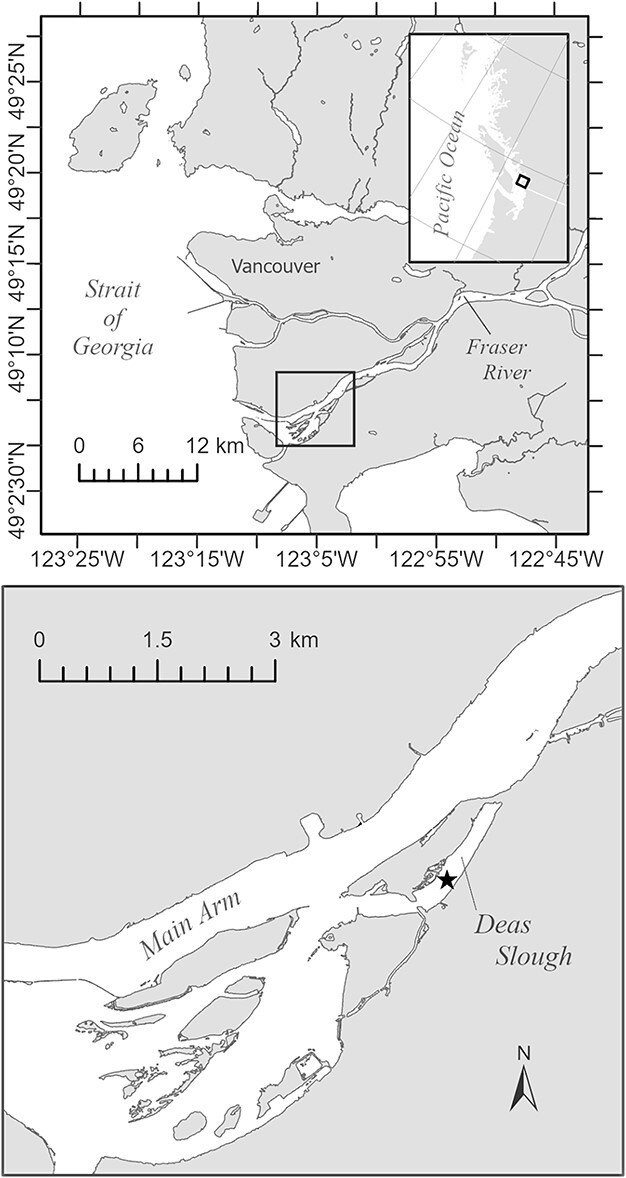
The Fraser River Estuary, Canada (upper panel). Station location along the South (Main) Arm of the estuary is indicated by a black star.

The FRE (Fig. 1) is classified as a moderately stratified or highly stratified salt wedge system, depending on river discharge and tidal range (Kostaschuk and Atwood, 1990). The Fraser River remains undammed leaving the estuary subject to an unmodified flow regime that is dominated by a snowmelt freshet that typically peaks mid-June (Morrison *et al.,* 2002). The position of the salt wedge in the estuary is controlled by river discharge and tidal height (Kostaschuk and Atwood, 1990). Along the South Arm of the estuary, maximum water column average salinities of ~ 13 have been reported to occur during the late winter–spring period of low river discharge (Breckenridge *et al.,* 2020). Minimum salinities (~ 1) occurred during the summer freshet. During the high river discharges typical of the freshet, all salt is flushed from the estuary on the greater ebb tide (Kostaschuk and Atwood, 1990; Jay *et al.,* 2007).

Water column average temperatures collected along the South Arm of the estuary have been reported to range from a low of ~ 5°C in January to a high of ~ 20°C in August (Breckenridge *et al.,* 2020). Deas Slough (49°07.07 N, 123°03.74 W) was chosen as the collection site for the present study because *E. affinis* were found to be abundant at this location (Breckenridge *et al.,* 2020) and *Eurytemora* nauplii have been hatched from the sediments (unpublished data). The slough is a former side channel that was dammed at the upstream end. Deas Slough has mean and maximum depths of approximately 5 and 10 m, respectively (Birtwell *et al.,* 1987). The presence of a sill results in a highly stratified water column and slows the loss of saline water during the freshet. Waters behind the sill can become hypoxic, particularly after the slough is cut off from the salt-wedge by high river discharge (Birtwell *et al.,* 1987).

### Environmental data and *Eurytemora* abundance

Environmental conditions and *Eurytemora* spp. abundance and population structure at the study site were estimated as part of a larger zooplankton monitoring program (August 2013–May 2016) described in Breckenridge *et al.* (2020). Water column properties were measured using casts of either an RBR XR-620 or a SeaBird SBE25 CTD and by collection of replicate surface water samples for chlorophyll analysis. Water samples were immediately filtered through Whatman GF/F filters, which were then kept on ice in the dark until returning to the laboratory, where they were stored in a − 20°C freezer. Chlorophyll pigments were extracted for 24 hours in 90% acetone and measured on a Turner Designs TD-700 fluorometer. Chlorophyll processing occurred within one week of sample collection. Statistics for daily discharge for the Fraser River, as measured in Hope, BC, Canada, were retrieved from Environment Canada at wateroffice.ec.gc.ca.

Daytime vertical tows of a 100-μm, 0.5-m diameter, conical net were collected near high tide, monthly to twice monthly from January 2015 to May 2016. Zooplankton samples were immediately preserved in 4% buffered formalin. Upon sample processing, *E. affinis* stages were identified according to Katona (1971) and Grice (1971). Samples with counts of fewer than 10 *Eurytemora* spp. were excluded from population structure statistics. Abundances of *E. affinis* may be overestimates due to the presence of *Eurytemora americana,* which is indistinguishable from *E. affinis* at younger copepodid stages (CI-CIII). We expect that this bias is slight because when CIV-CVI *E. americana* are present (November–May), they are at much lower abundance than *E. affinis*. The abundance of *E. affinis* nauplii was estimated for a subset of samples (May–October 2015) by examining copepod nauplii at 1000x magnification. In sum, 40–60 individual nauplii were examined for most dates, though the number was far greater when *E. affinis* abundance was very low. Note that the mesh size (100 μm) was too large to provide accurate estimates of naupliar abundance. We therefore use these estimates only to get a rough idea of naupliar dynamics.

### Collection of individuals and water

Adult females of *E. affinis* were collected from Deas Slough monthly during February 2015–May 2015, August 2015–October 2015 and February 2016–May 2016 monitoring surveys using gentle tows of a 1-m mouth, 100-μm conical net, and were diluted into a bucket with water from the collection site. Live specimens and water were kept in coolers and were returned to the laboratory within 2–3 hours. Ambient water for the incubations was obtained from the halocline depth (~5 m) using a Niskin bottle and was filtered through a 63-um mesh sieve to remove zooplankton and eggs.

### Incubations

Live zooplankton were sorted immediately upon return to the laboratory. Adult *E. affinis* females that were undamaged and displayed a normal level of activity were picked using a wide-mouth pipette under a Leica S8 APO stereomicroscope. Both ovigerous and non-ovigerous females were included in incubations. We aimed for each set of incubations to include 30 females, though this number was decreased in some incubations due to insufficient collection of *E. affinis* and/or to mistaken incubation of the congener, *E. americana*. Females were rinsed and isolated using a welled Plexiglas® plate and the presence of eggs and spermatophores noted and their number estimated under the stereomicroscope.

Adult females of *E. affinis* were incubated individually in 250-mL Nalgene™ bottles filled with 63-μm pre-screened water collected from the sample site. The relatively large incubation volume was used to reduce the potential cannibalism of nauplii by adults, which can result in underestimation of egg production (Runge and Roff, 2000). Bottles were incubated in temperature-controlled rooms, where temperatures were set to approximate natural conditions (Table I). Incubation durations ranged from 18 to 24 hours. At the end of the incubation, females were inspected to ensure that they were alive, filtered over a 20-μm sieve, along with the contents of the incubation bottle, and then preserved in 4% formalin. Upon processing, female prosome length and width were measured under a stereomicroscope equipped with a micrometer, and clutches, if present, were removed and dissected to provide an accurate final clutch size. On rare occasions, clutches contained one to two eggs that were gray or degraded in appearance. These eggs were assumed to be nonviable and were not counted. Accompanying water was scanned and any *Eurytemora* nauplii, released unhatched eggs, and egg casings enumerated. Multiple egg types were not apparent from light microscopy, however Ban (1992) has previously noted that subitaneous and diapause eggs of *E. affinis* cannot be distinguished via light microscopy. *Eurytemora affinis* is an egg-carrying copepod and, to our knowledge, does not drop subitaneous eggs. Based on Mauchline (1998), Glippa *et al.* (2011), and from personal observation, *Eurytemora* nauplii hatch from eggs that remain attached to females. We therefore considered released eggs that were healthy in appearance (i.e. not gray, degraded or crumpled) to be resting eggs.

**Table I:** Summary of environmental conditions at the time of collection (salinity range, temperature range (°C), minimum oxygen (ml L^−1^) recorded from bottom water, and daylength (hours)) and incubation (salinity, temperature (°C)) of Eurytemora affinis from Deas Slough, BC, and incubation time (hours)

	Collection conditions				Incubation conditions		
Date	Salinity range	Temperature range	Oxygen minimum (ml L^−1^)	Daylength (hours)	Salinity	Temperature	Time (hours)
2015-02-12	0.3–8.9	4.9–6.7	2.5	10	5.0	4	24
2015-03-17	0.6–9.5	6.7–7.5	4.6	11.7	8.8	4	24
2015-04-28	0.3–4.0	9.0–10.6	1.1	14.5	1.7	12.5	21
2015-05-12	0.1–0.4	11.8–13.2	6.3	15.2	0.3	12.5	23
2015-08-05	1.0–3.2	18.8–21.3	1.8	14.5	0.3	19	24
2015-09-03	0.1–7.8	16.8–18.2		13.3	5.2	19	21
2015-10-07	1.9–7.1	13.2–13.5		11.3	5.0	12.5	21
2016-02-17	0.3–14.7	5.8–6.5	6.4	10.3	5.5	12.5	22
2016-03-15	0.4–14.0	6.0–7.0	0.7	11.9	6.9	4	22
2016-04-20	0.2–13.4	7.5–11.8	0.3	14.1	9.5	12.5	22
2016-05-03	0.1–13.2	7.8–13.4		14.8	5.5	13	20
2016-05-17	0.1–13.0	8.4–14.0	1.1	15.5	5.2	13	18

We conducted additional incubations aimed at collecting live resting eggs for further culturing on two dates. In May 2015, six ovigerous CVI females were individually incubated and five CV females were incubated as a group with adult males in an attempt to collect resting eggs. In April 2016, 14 ovigerous females were incubated together in 500 mL of filtered estuarine water. Females were checked daily until they no longer held clutches (2–3 days) and then the incubation water was scanned via stereomicroscopy for resting eggs and nauplii.

### Estimation of daily egg production rate (EPR) and resting egg production

A comparison of estimates of clutch size (*CS*) of 171 females before and after dissection showed thar our CS estimates decreased in accuracy from near perfect for CS < 16 to ±6 eggs at CS > 60 eggs. The comparison also revealed that we slightly underestimated the actual egg count. A corrective equation was calculated using clutch size estimates and dissection counts from 127 females with clutch sizes > 16 and applied to CS estimates, where the initial clutch size, *CS_i_* = estimated *CS*/0.9676–0.3811. This correction adds less than 1 egg for clutch size estimates ≤ 40. Daily EPR was calculated as


$$ \mathrm{EPR}=\left({CS}_f+{N}_e+{N}_n-{CS}_i\right)/\Delta \mathrm{t} $$

where *N_e_* is the number of released eggs, *N_n_* is the number of nauplii, and *CS_f_* and *CS_i_* are the final and initial clutch sizes, respectively. Negative EPRs were corrected to zero and, to be conservative in estimates of resting eggs all missing progeny were assumed to be nauplii. Daily EPR was calculated as the average of individual EPR estimates, including those of females that did not produce eggs. The rate of resting egg production (eggs female^−1^ day^−1^) was calculated by dividing the number of released eggs by incubation time.

### Statistical analysis

We investigated the relationship between the occurrence of resting eggs and environmental conditions using beta regressions, which are suitable for the analysis of proportion data (Douma and Weedon, 2019). The proportion of eggs that were released by individual incubated females was calculated by dividing the number of released unhatched eggs present at the end of the incubation by the total number of offspring. The number of offspring was considered to be either the sum of resting eggs and nauplii present at the end of the incubation or the difference between the initial egg count and the final egg count, whichever was greater. Eggs with unknown fates were assumed to have resulted in nauplii. Female-specific proportions were then averaged resulting in an average proportion resting eggs for each incubation date. Our data included two zero values and were thus compressed using


$$ y=\left[{y}_i\ \left(n-1\right)+0.5\right]/n $$

where *n* is the number of observations, to fit within the (0,1) interval assumed by beta regression (Smithson and Verkuilen, 2006). Beta regression models, using logit linkages, were fit for mean water column temperature, mean water column salinity, 7-day mean of daily river discharge from the previous week as measured at Hope, BC, and surface chlorophyll *a* concentration. This analysis was conducted using the betareg R package (Cribari-Neto and Zeileis, 2010) following Douma and Weedon (2019). All data analyses were performed in R version 4.4.0 (R Core Team, 2024).

## RESULTS

### Environmental conditions

**Fig. 2:**
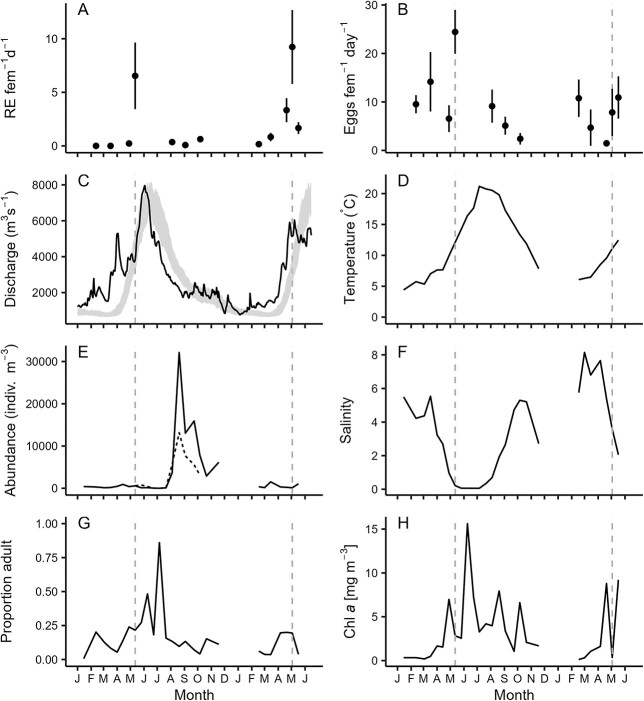
Egg production of *Eurytemora affinis* adult females and environmental conditions over the study period (February 2015–May 2016). (**A**) Average daily number of resting eggs (RE female^−1^ day^−1^ ± 1 SE) per *E. affinis* female. (**B**) Egg production rate (eggs female^−1^ day^−1^ ± 1 SE), and environmental conditions over the study period (February 2015–May 2016). (**C**) River discharge (m^3^ s^−1^) for the Fraser River measured at Hope, BC, presented with a band representing interquartile range of daily discharge values (1912–2014) using data retrieved from wateroffice.ec.gc.ca. (**D**) Water column average temperature (°C) and (**F**) salinity, and (**H**) surface water chlorophyll *a* concentration (mg m^−3^) at Deas Slough in the FRE, BC. (**E**) Average abundance (individuals m^−3^) of *E. affinis* copepodids (solid line) and abundance of nauplii (dashed line) stages collected at Deas Slough in the FRE, BC, January 2015–May 2016 and May 2015–October 2015, respectively, and (**G**) proportion of copepodids that were adult. Note that naupliar abundance is underestimated and is presented to give an indication of dynamics only. Dashed vertical gray lines in (B)–(H) indicate the date of peak observed resting egg production in both 2015 and 2016. There were no cruises in December 2015 or January 2016.

Salinity conditions in Deas Slough during spring 2015 and 2016 varied considerably. The FRE experienced record-breaking high river discharge in spring 2015 (Fig. 2C). Salinity at the collection site is typically stratified and the presence of a sill at the inlet retains salt in the slough even as river discharge increases. In 2015, salinity at depth began to decrease after mid-April from a high of ~ 9, with most salt being flushed from the slough by mid-May (Fig. 2F). The salt wedge again began to reach the slough in late July, as the freshet tapered. In 2016, which was more typical with respect to river discharge, the freshet began later and while average salinity again began to decrease in April, brackish bottom waters (~ 13) remained in the slough through the end of our monitoring program in May 2016.

Temperature at the collection site was relatively homogeneous, with typically less than a 1°C difference between surface and bottom waters. Average water temperatures at the collection site peaked in July (21°C) and were at a minimum (4–6°C) in winter and early spring (Fig. 2D). Temperatures were similar during both spring periods. Surface water chlorophyll concentration was variable but generally elevated May–October (Fig. 2H).

### Abundance and population structure

*Eurytemora affinis* were present in the water column year-round in Deas Slough (Fig. 2E). In 2015, *E. affinis* copepodid abundance increased slowly to a low peak in mid-April (921 individuals m^−3^ ± 431 SE), then decreased to a minimum on July 6 (8 individuals m^−3^ ± 3 SE), when mean water column salinity and temperature were < 0.1 and 21°C, respectively. Minimum abundances coincided with a copepodid population dominated by adults (Fig. 2G). The population then increased rapidly and an abundance of 35 155 individuals m^−3^ ± 9 385 SE was recorded on 20 August 2015, while mean water column temperature remained near its seasonal peak (20°C) and salinity was 2. Peak copepodid abundances, often exceeding 10 000 individuals m^−3^, occurred August–November. In spring 2016, copepodid abundance was again low, with a peak abundance of ca. 1500 individuals m^−3^ recorded in mid-March.

Changes in naupliar abundance (underestimated and investigated only for the period of May–October 2015, as described above) tracked changes in copepodid abundance. For one of these dates (6 July 2015), we examined 337 copepod nauplii but did not observe any *E. affinis*, which suggests very low abundance of *E. affinis* nauplii for that date (< 8 individuals m^−3^).

### Resting egg production and daily EPR

We incubated a total of 335 *E. affinis* females over 12 dates. Resting eggs were noted on multiple dates and their occurrence peaked in April 2016 and in May incubations of both years (Table II). During this period, estimates of the rate of Resting egg production ranged between 1.7 and 9.2 female^−1^ day^−1^ (Fig. 2A). In the May 2015 incubation, 87.5% of females with clutches at the onset of the incubation released eggs with one individual releasing 77 eggs. During the 3 May 2016, incubation, 72% of females with clutches released eggs with the largest release being of 63 eggs. Resting eggs often remained loosely adhered to each other. Clutches often resulted in both nauplii and resting eggs. Females that released eggs typically carried spermatophores and the proportion of incubated females with attached spermatophores was similar to or higher than those of incubations occurring at other times of the year (Table II).

**Table II:** Summary of characteristics of incubated Eurytemora affinis.

Date	*N*	*F_clutch_*	*PL* ± SD (mm)	*CS_x_* ± SD	*F_sperm_*	*F_drop_*	Max. drop
2015-02-12	40	67.5	0.79 ± 0.05	29 ± 9	20.0	0.00	0
2015-03-17	10	80.0	1.00 ± 0.07	36 ± 12	20.0	0.00	0
2015-04-28	29	72.4	0.97 ± 0.04	44 ± 15	44.8	13.8	3
2015-05-12	30	53.3	0.97 ± 0.03	47 ± 19	53.3	46.7	77
2015-08-05	30	85.7	0.86 ± 0.05	39 ± 23	46.7	16.7	3
2015-09-03	29	79.3	0.76 ± 0.04	11 ± 7	58.6	3.6	2
2015-10-07	29	65.5	0.74 ± 0.03	6 ± 3	41.4	20.7	5
2016-02-17	29	63.0	0.91 ± 0.06	45 ± 23	29.6	7.4	3
2016-03-15	20	70.0	0.97 ± 0.04	40 ± 17	10.0	30.0	6
2016-04-20	27	74.1	0.93 ± 0.04	27 ± 14	39.3	48.2	18
2016-05-03	30	63.3	0.94 ± 0.04	61 ± 35	46.7	43.3	63
2016-05-17	30	50.0	0.91 ± 0.04	39 ± 26	36.7	33.3	8

The number of females incubated (*N*), the percentage of incubated individuals that carried eggs (*F_clutch_*) and had attached spermatophores (*F_sperm_*) at the beginning of the incubation, mean prosome length (*PL* ± 1 SD) in mm, mean clutch size (*CS* ± 1 SD), percentage of individuals that released unhatched eggs (*F_drop_*) and the largest recorded egg release (Max. drop).

The production of eggs in the incubations was variable with the highest percentage of females (80%) extruding eggs during the March 2015 incubation and the lowest percentage (21%) during the 3 May 2016 incubation. Our estimates of EPR ranged from a minimum of 1.5 eggs female^−1^ day^−1^ (± 0.5 SE) in April of 2016 to a maximum of 24.4 eggs female^−1^ day^−1^ (± 5.1 SE) in May 2015 (Fig. 2B), both dates for which we recorded a relatively high number of resting eggs (Fig. 2A). Prosome lengths and clutch sizes were largest during spring incubations and comparable across 2015 and 2016 (Table II). EPR decreased in autumn and coincided with an observed decrease in average clutch size and female prosome length (Table II).

The separate incubations to collect live resting eggs yielded only a single released egg and many nauplii. The egg was not monitored further. These incubations were flawed in that, while females were checked to determine whether they continued to carry eggs, no check was made for resting eggs until the incubations were terminated a few days later. We therefore cannot exclude the possibility of resting eggs that hatched before we terminated the incubation or the possibility that resting eggs were cannibalized.

**Fig. 3:**
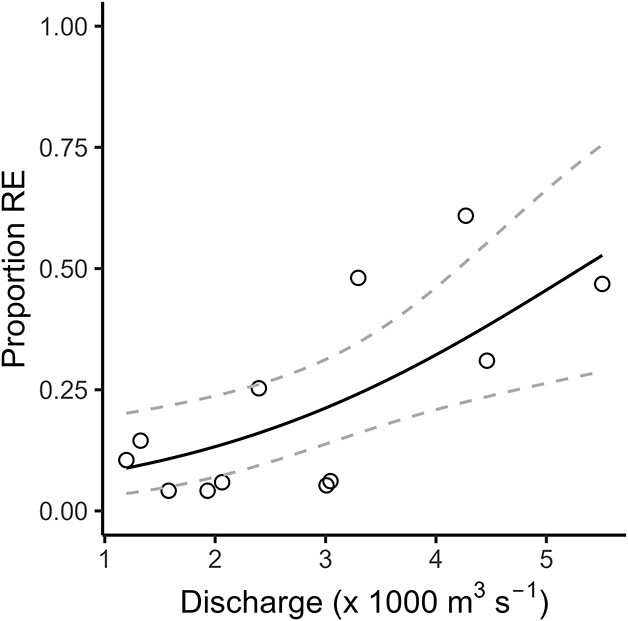
Mean proportion of *Eurytemora affinis* offspring that were released as unhatched eggs (RE), rather than nauplii, increased with the 7-day mean of river discharge (× 1000 m^3^s^−1^) as measured by Environment Canada at Hope, BC, Canada (wateroffice.ec.gc.ca). Original data points plotted as circles. Dashed lines show upper and lower 95% confidence limits.

Results of beta regression analysis indicated that a higher proportion of offspring were released as eggs rather than nauplii when river discharge was high (β = 0.57, *z* = 3.43, *P* < 0.001, *Pseudo-R^2^* = 0.48, Fig. 3). Relationships were not detected with other investigated variables (salinity, temperature and chlorophyll *a* concentration).

## DISCUSSION

The incubations revealed that *E. affinis* released unhatched eggs during the spring, and that the proportion of offspring that were released as resting eggs increased with river discharge (Figs 2 and 3). Eggs released in Deas Slough would have sunk to the oxygen-deplete environment that exists in its bottom waters after it is cut off from the salt wedge by the freshet (Birtwell *et al.,* 1987). Both burial in sediment and anoxic conditions have been shown to inhibit hatching (Uye, 1980; Ban and Minoda, 1992). The re-entry of the salt wedge as the freshet receded would have increased salinity and potentially re-suspended the eggs, stimulating hatching. These nauplii likely contributed to the rapid increase in *E. affinis* that occurred in late summer (Breckenridge, 2022).

Copepod resting eggs are typically categorized as being either quiescent or diapause (Uye, 1985; Dahms, 1995). Quiescent eggs are subitaneous eggs that become dormant in direct response to adverse conditions and will resume development immediately after the return of favorable conditions (Danks, 1987). In contrast, diapause eggs are produced in anticipation of adverse conditions and undergo a refractory period before hatching. Because *E. affinis* were known to produce diapause eggs (Naess, 1991; Ban, 1992), we failed to consider the possibility of the species producing quiescent eggs or that resting eggs could hatch during the incubations. The hatching of released eggs could explain the discrepancy when comparing the outcomes of the May 2015 and April 2016 EPR incubations, where nearly half of females released unhatched eggs (Table II), with their concurrent, longer duration, incubations to collect live eggs, which yielded a single released egg and many nauplii. Glippa *et al.* (2014) have suggested that in the Seine estuary, France, *E. affinis* resting eggs may be either quiescent or diapausing. The disagreement between the two types of incubation in our study suggests that the resting eggs may have been quiescent or delayed hatching eggs (diapause eggs with a short refractory period; Chen and Marcus, 1997). However, a more rigorous investigation is warranted before categorizing these eggs.

The healthy appearance of the released eggs, which were indistinguishable from subitaneous eggs, suggests that the released eggs were viable. Nonviable eggs have been noted to differ noticeably in appearance from viable eggs (Ban, 1992). Further, while the extrusion of clutches of nonviable eggs has been observed under laboratory conditions in the absence of males (Katona, 1975; Ban, 1992; Glippa *et al.,* 2013), the high percentage of females carrying spermatophores in the incubations suggests that females were not mate-limited (Table II). Lasley-Rasher *et al.* (2014) suggested that carrying unfertilized eggs should be rare under natural conditions as egg-carrying increases the risk of predation for egg-brooding females (Hairston Jr. *et al.,* 1983; Winfield and Townsend, 1983; Svensson, 1995).

An alternative purpose to the early release of clutches by egg-carrying copepods is provided by Koski *et al.* (2014), who suggest that it increases reproductive output by allowing females to produce the subsequent clutch more rapidly. They observed an abundance of loose clutches of the sac-spawner *Microsetella norvegica* in a Greenland fjord and inclusion of those loose clutches of eggs into EPR estimates was necessary to account for the high abundances of this species. During the present study, *E. affinis* eggs were released during a period when overall EPR was relatively high and prosome lengths and clutches were relatively large, suggesting that females were not food-limited (Ban, 1994; Lloyd *et al.,* 2013). It is plausible that the time required for eggs to develop at observed temperatures (3–4 days) could limit their reproductive output during this period. However, *E. affinis* population structure shifted toward late stage copepodids following the period of releasing eggs (Fig. 2), which is consistent with the eggs not hatching immediately following detachment. Given that the release of eggs occurred just prior to the seasonal collapse of the population (Fig. 2), for these to remain subitaneous and hatch would suggest a large waste of reproductive effort.

*Eurytemora affinis* resting eggs are generally considered as an overwintering mechanism (Glippa *et al.,* 2013; Holm *et al.,* 2018); however, we were only able to find two studies which investigated the timing of resting egg production in the field. A freshwater population of *E. affinis* in Lake Ohnuma, Japan, produced diapause eggs in autumn (Ban and Minoda, 1991 as cited in Ban, 1992) in response to decreased photoperiod (Ban, 1992). Based on the presence of eggs in the sediment and the seasonal pattern of abundance and population stage structure, *E. affinis* also produced diapause eggs for overwintering in the Baltic Sea (Katajisto *et al.,* 1998). Our results do not preclude the possibility of resting egg production in autumn or winter; however, the presence of a second, higher peak of *E. affinis* in the late summer and autumn suggests that springtime resting egg production by the FRE population does not serve the purpose of overwintering. As *E. affinis* is a species complex (Lee, 2000), within which optimal conditions and vital rates can vary significantly (Beyrend-Dur *et al.,* 2009; Devreker *et al.,* 2012), resting egg production could be expected to differ between clades or even between populations from differing estuaries (Glippa *et al.,* 2013), as has been found for species of *Acartia* (Uye, 1985; Avery, 2005; Drillet *et al.,* 2011). Springtime and summer resting egg production has been suggested previously for *E. affinis* in the Seine, based on the abundance of nauplii hatching from the sediment in the summer (Glippa *et al.,* 2014). Springtime resting egg production in *E. affinis* has also been reported from a laboratory study where production of diapause eggs occurred in response to accumulation of their own metabolites, which may have served to avoid competition and resulting food shortage (Ban and Minoda, 1994). We consider crowding an unlikely cause of springtime resting egg production in the FRE because resting eggs were produced when copepodid densities were low, < 1 200 individuals m^−3^, in comparison to peak summer/autumn densities, which exceeded 30 000 individuals m^−3^.

Springtime production of resting eggs is typically attributed to avoidance of high temperatures and has been documented in many coastal calanoid copepods (Uye, 1985; Sullivan and McManus, 1986; Chen and Marcus, 1997; Castro-Longoria and Williams, 1999; Castellani and Lucas, 2003; Berasategui *et al.,* 2012; Holm *et al.,* 2018). During the present study, the highest rate of resting egg production was observed when mean water temperatures were in the range of 11–12°C. We do not know the thermal tolerance of *E. affinis* in the FRE, but populations from other estuaries have been raised at temperatures up to 25°C (Heinle and Flemer, 1975; Poli and Castel, 1983). Aestivation seems unlikely for *E. affinis* in the FRE, given that the period of most rapid *E. affinis* population growth coincided with the period of peak water temperatures (July/August). Predator avoidance was the cited cause of springtime diapause egg production in the freshwater calanoid copepod, *Diaptomus sanguineus* (Hairston *et al.,* 1983). This study did not assess predator abundance so we cannot exclude this possibility.

With respect to timing of resting egg production, the most proximate of “catastrophes” is the seasonal peak of river discharge, the freshet. The seasonal predictability of high river discharge in snowmelt-dominated river basins would allow copepods to use correlated environmental cues as a predictor of future adverse conditions (Dahms, 1995). It has been suggested that, in estuarine areas, benthic resting eggs could help endemic copepod populations avoid flushing (Uye, 1980; Runge and Simard, 1990; Newton and Mitchell, 1999). The estuary of the Fraser River is fast-flowing, and residence times in the main channel have been estimated to be 6–30 hours, depending on level of river discharge (Ages and Woollard, 1988). During the freshet, the salt-wedge is flushed from the estuary on a daily basis (Kostaschuk and Atwood, 1990), which means that, unlike in many other estuaries, there is no downstream refuge available to this population during high river discharge. These conditions can be expected to pose great challenges to an estuarine copepod, both with respect to position maintenance and osmoregulation. Strong tidal vertical migration (TVM) behavior in local *E. affinis* is evidence that the threat of advection has shaped this population (Breckenridge, 2022). TVM is likely sufficient to retain *E. affinis* in Deas Slough due to the sill; however, in the larger estuary this is likely not the case given the increase in currents associated with the freshet. In areas where TVM is sufficient for retention, *E. affinis* would still need to survive and develop in fully fresh waters. While *E. affinis* has invaded freshwaters on multiple occasions (Lee, 1999), this species has not been found in samples from freshwater stations in the FRE. The coincidence of resting egg production in the population with the freshet, during a period when EPR was relatively high and conditions could be considered as encouraging for growth (moderate temperatures, low population density, relatively high chlorophyll; Fig. 2), suggests that resting egg production may occur either in anticipation of the low salinity and/or high current velocity conditions associated with the freshet, or in direct response to conditions associated with the freshet.

The present study did not attempt to identify proximate cues for resting egg production; however, several potential cues/triggers warrant mention. Hansen (2019) suggested low oxygen concentration as a trigger for subitaneous eggs to enter quiescence. Low oxygen conditions exist in the bottom waters of Deas Slough during the period of resting egg production (Birtwell *et al.,* 1987) and female *E. affinis* have been shown to spend much of their time in bottom waters of the slough (Breckenridge, 2022). Photoperiod, and photoperiod modified by temperature, are the most commonly cited proximate cues of seasonal diapause egg production in calanoid copepods (Marcus, 1980; Uye, 1985; Hairston Jr and Kearns, 1995; Baumgartner and Tarrant, 2017; Hansen, 2019; Takayama and Toda, 2019) and decreased photoperiod has specifically been found to cue diapause egg production in *E. affinis* (Ban, 1992; Glippa *et al.,* 2013). While *E. affinis* in the FRE released resting eggs when photoperiod was increasing, perceived photoperiod may have decreased due to the rapid increase in suspended sediment that occurs at the beginning of the freshet (Kostaschuk *et al.,* 1989). In this way, both reduction in photoperiod and decreasing oxygen in bottom waters could be reliable cues of increasing river discharge in Deas Slough. In the greater estuary, however, silled sloughs are rare and bottom waters would remain well-oxygenated throughout the freshet. Finally, low food quality has been found to stimulate resting egg production (Drillet *et al.,* 2011). Food quality was not assessed during this study but relatively high concentrations of chorophyll *a* (Fig. 2H), paired with high EPR, large prosome lengths, and large clutch sizes during spring incubations (Table II), do not suggest that food limitation was occurring here.

Understanding both the adaptive value of resting eggs and cues for their production is complicated by recent physical modifications made to the estuary. Our study was based in Deas Slough, a former side channel that was dammed in 1948, because *E. affinis* were largely restricted to slough environments (Breckenridge *et al.,* 2020). It is therefore possible, given the flexibility of resting egg production (Glippa *et al.,* 2013; Hansen, 2019), that the occurrence and timing of resting egg production in Deas Slough differs from that in the larger estuary.

## CONCLUSIONS

The results of this study suggest that *E. affinis* in the FRE produced resting eggs as river discharge was increasing in the spring. Resting egg production by *E. affinis* is typically an overwintering mechanism (Katajisto *et al.,* 1998; Glippa *et al.,* 2013; Holm *et al.,* 2018) but we suggest that, in the FRE population, the ultimate driver is avoidance of flushing and/or low salinities. Given the extent to which modern river hydrographs have been altered through damming, water withdrawals, and changing climate (Haddeland *et al.,* 2014), the possibility of estuarine copepods producing resting eggs for estuarine retention has important ramifications. Many estuaries no longer experience historical peak flows, and for others, such as the Fraser, the timing of peak flows is changing (Morrison *et al.,* 2002), which raises the question of how modification of the hydrograph might affect the life history of these populations. In this case, determining whether *E. affinis* resting eggs are quiescent or diapausing and understanding what cues their production is necessary to predict how the *E. affinis* population will respond to changes in the annual hydrograph. If the eggs that are released are quiescent, this would suggest that the timing of production would shift with the hydrograph, whereas if they are diapausing, timing of production may be less flexible, which could result in decreased contributions to the egg bank with the early onset of the freshet.

## Data Availability

Data that support the findings of this study are available from the corresponding author, J.K.B., upon request.
